# Meal-Related Asprosin Serum Levels Are Affected by Insulin Resistance and Impaired Fasting Glucose in Children With Obesity

**DOI:** 10.3389/fendo.2021.805700

**Published:** 2022-01-06

**Authors:** Domenico Corica, Giorgia Pepe, Tommaso Aversa, Monica Currò, Selenia Curatola, Alessandra Li Pomi, Angela Alibrandi, Riccardo Ientile, Malgorzata Wasniewska

**Affiliations:** ^1^ Department of Human Pathology of Adulthood and Childhood “G. Barresi”, University of Messina, Messina, Italy; ^2^ Department of Biomedical and Dental Sciences and Morpho-Functional Imaging, University of Messina, Messina, Italy; ^3^ Department of Economics, University of Messina, Messina, Italy

**Keywords:** asprosin, adipokine, glucose homeostasis, childhood obesity, insulin resistance, children

## Abstract

Asprosin physiologically increases in fasting conditions and decreases with refeeding and has been implicated in glucose homeostasis. An alteration of meal-related circadian oscillation of asprosin has been suggested in adults affected by type 2 diabetes mellitus.

Aims of this study were to test the hypothesis of an alteration in the meal-related variation of asprosin levels in non-diabetic children and adolescents with obesity and to assess which metabolic variables condition this variation in non-diabetic children and adolescents with obesity. This is a cross-sectional study which included 79 children and adolescents with obesity. Children underwent clinical and biochemical assessments, including oral glucose tolerance test (OGTT), and liver ultrasound evaluation. Asprosin serum levels were measured by an enzyme-linked immunosorbent assay at a fasting state and at the 120-minute OGTT timepoint (2h-postprandial asprosin). Fasting and 2h-postprandial asprosin serum levels did not significantly differ in the entire study population (374.28 ± 77.23 vs 375.27 ± 81.26;p=0.837). 55.7% of patients had a significant increase in 2h-postprandial asprosin compared with fasting levels. The asprosin level increase condition was significantly associated with HOMA-IR (OR,1.41; 95%CI,1.005-1.977; p=0.047), fasting glycaemia (OR,1.073; 95%CI,1.009-1.141;p=0.024) and HOMA-B (OR,0.99; 95%CI,0.984-0.999; p=0.035). Moreover, the IFG condition was associated with the increase in asprosin levels (OR, 3.040; 95%CI, 1.095-8.436; p=0.033), even after adjustment for HOMA-IR, BMI SDS, sex and pubertal stage. Insulin resistance and IFG influence meal-related changes of asprosin serum levels in our study population of obese, non-diabetic, children. Alteration of asprosin circadian secretion might be an early biomarker of impaired glucose regulation in obese children with insulin resistance.

## Introduction

Childhood obesity represents one of the most important health issues worldwide and is associated with an increased risk of metabolic complications, such as insulin resistance (IR) and impaired glucose regulation (IGR), including impaired fasting glucose (IFG) and impaired glucose tolerance (IGT) (1, 2). IR has become a common feature of childhood obesity being directly related to adiposity (3). Adipose tissue is known to play an important role as an endocrine organ secreting several adipokines involved in the pathogenesis of obesity-related IR (1).

Asprosin is a recently identified adipokine, produced mainly by white adipose tissue (4), implicated in the pathophysiology of several conditions, such as obesity, insulin resistance, type 2 diabetes mellitus (T2DM), and cardiovascular diseases, by preclinical and clinical studies (5, 6).

Previous studies described asprosin involvement in glucose homeostasis as consisting of appetite regulation through orexigenic AgRP+ neurons and promotion of hepatic gluconeogenesis under fasting conditions (4, 7). Accordingly, asprosin serum levels increase in fasting conditions and decrease with refeeding in physiological conditions (4).

Several studies have documented a positive correlation between asprosin levels and HOMA-IR (8–10) while others have not confirmed this correlation (6) or have even found a negative correlation (11). Elevated serum asprosin concentrations have been documented in subjects affected by T2DM compared to healthy controls (10, 12, 13). In addition, an alteration of meal-related circadian oscillation of asprosin serum levels has been reported in T2DM patients compared to non-diabetic controls (14).

Based on these findings, although partly contrasting, asprosin seems to play a role in glucose homeostasis regulation, but no data are available on meal-related asprosin level changes in children and adolescents with obesity.

Aims of this single-center cross-sectional study were to test the hypothesis of an alteration in the meal-related variation of asprosin levels in non-diabetic children and adolescents with obesity and to assess which metabolic variables condition this variation.

## Materials and Methods

This is a single-center, cross-sectional study carried out from October 2020 to May 2021. Inclusion criteria were age between 5 and 16 years; BMI ≥ +2 standard deviation score (SDS), in accordance with definition of obesity by the World Health Organization (WHO) for children from the age of 5 years; Caucasian ethnicity; born as healthy full-term infant adequate for gestational age. Exclusion criteria were genetic and/or endocrine causes of obesity; diabetes; chronic diseases; chronic pharmacological therapies; smoking. All procedures were performed in accordance with the Declaration of Helsinki and were approved by the Ethics Committee of Messina (N.552-17/04/2019). Written informed consent was obtained from all parents or legal tutors.

### Clinical and Biochemical Evaluation

At recruitment, physical evaluation was performed according to standardized procedures, including assessment of height, weight, BMI, BMI SDS, waist circumference (WC), WC-to-height ratio (WHtR), systolic and diastolic blood pressure (15). Pubertal stage was determined according to the Tanner classification (15); patients were considered in a pubertal stage from Tanner stages B2 for females and G2 for males. Children underwent fasting biochemical assessment (lipid profile, thyroid, kidney, liver function tests, and oral glucose tolerance test (OGTT)), as previously described (16). OGTT was performed according to a standard procedure (1.75 g/kg body weight, up to a maximum of 75 g) according to the American Diabetes Association (ADA) guidelines (17), with sampling at 0, + 30, +60, +90, +120 minutes for measurements of glucose and insulin. IFG was defined as fasting plasma glucose between 100 and 125 mg/dL and IGT as plasma glucose level 2 hours after a 75-g glucose load (OGTT), which was between 140 and 199 mg/dL (17). Blood samples for the serum asprosin assay were taken after fasting at the beginning of the OGTT, after at least 8 hours of overnight fasting, and at the 120-minute OGTT timepoint (2h postprandial asprosin). Asprosin serum levels were measured using an enzyme-linked immunosorbent assay (ELISA) kit accordingly to manufacturer’s instructions (MyBioSource, USA; catalog number:MBS9716571). The detection threshold was 1 pg/mL and no significant cross-reactivity between human asprosin and analogues was reported; the intra-assay and inter-assay coefficient of variation (CV) values were <9% and <11%, respectively. Asprosin concentrations were expressed as pg/mL.

Homeostasis model assessment of insulin resistance (HOMA-IR), homeostasis model assessment of β-cell function (HOMA-B), Matsuda-index, insulinogenic-index (IGI) were calculated, as previously detailed (18). Areas under the curves for glucose (AUCg) and insulin (AUCi) and their ratio were also evaluated. IR was defined as HOMA-IR > 2.5 in prepubertal children and > 4 in pubertal subjects (16). Seventy-one patients underwent hepatic ultrasound (US) assessment. Diagnosis of liver steatosis was made by conventional liver US depending on the presence of at least two of the following abnormal findings: 1) diffusely increased echogenicity of the liver compared with kidney or spleen; 2) US beam attenuation; 3) poor visualization of intrahepatic structures (19, 20).

### Statistical Analysis

Numerical data were expressed as mean and SDS and categorical variables as absolute frequency and percentage. The non-parametric approach was used since most numerical variables were not normally distributed, as verified by the Kolmogorov-Smirnov test. The Wilcoxon test for dependent samples was applied to identify possible significant differences between fasting and 2h-postprandial glycaemia, insulin and asprosin, both in the entire study population and in subgroups defined according to sex, pubertal stage, BMI SDS (BMI SDS ≤ or > 2.5) and the presence of IR, IFG and hepatic steatosis. To compare clinical and metabolic parameters between the two groups of patients identified according to meal-related asprosin variation the Mann-Whitney test (for numerical parameters) and the Chi Square test (for categorical variables) were applied. The Spearman correlation test was used to assess the existence of significant interdependence between asprosin levels (both fasting and 2h-postprandial levels) and clinical and biochemical parameters; in addition, partial correlation was also estimated in order to check for sex, pubertal stage and BMI SDS. A stepwise multiple logistic regression analysis was carried out to identify which metabolic predictors significantly affect the variation of asprosin levels considering the dichotomous variable asprosin increase/non-increase between fasting and 2h-postprandial status, through the estimation of different models (Model 1: age, sex, BMI SDS, HOMA-IR, HOMA-B, IGI, Matsuda index, AUC_i_/AUC_g_-ratio. Model 2: age, sex, BMI SDS, fasting and 2h-postprandial glycaemia, fasting and 2h- postprandial insulin. Model 3: age, sex, BMI SDS, Triglycerides/HDL-ratio, Total Cholesterol/HDL-ratio. Model 4: age, sex, BMI SDS, Triglycerides, LDL, HDL, Total Cholesterol. Model 5: age, sex, BMI SDS, alanine aminotransferase (ALT), aspartate aminotransferase (AST), gamma-glutamyl transferase (GGT), hepatic steatosis). In addition, further logistic regression models were estimated to assess the influence of IFG on the asprosin level increase condition, even after adjustment for HOMA-IR, BMI SDS, sex and pubertal stage (Model 1: crude model; Model 2: HOMA IR; Model 3: HOMA IR, BMI SDS; Model 4: HOMA IR, BMI SDS, sex; Model 5: HOMA IR, BMI SDS, sex, pubertal stage); in this analysis, pubertal stage, rather than age, was chosen as an independent variable because of the well-known significant implications of puberty on metabolic changes.

Statistical analyses were performed using IBM SPSS for Windows, Version 22 (Armonk, NY, IBM Corp.). A p-value < 0.05 was considered to be statistically significant.

## Results

Seventy-nine children (aged 11.5 ± 2.6 years) were consecutively recruited, 40 males and 39 females; 64.5% of them were in a pubertal stage. Clinical and biochemical characteristics of the study population are shown in **Table 1**. 67% had IR, 33% had IFG and 8.8% IGT. No patient was diagnosed with T2DM. 39% of patients were diagnosed with hepatic steatosis.

**Table 1:** Clinical and biochemical features of study population.

	Mean	SDS
**Age (years)**	11.46	2.61
**Weight SDS**	2.25	0.58
**Height SDS**	0.48	1.18
**BMI**	29.49	4.33
**BMI SDS**	3.16	0.93
**WC (cm)**	89.50	10.29
**WHtR**	0.59	0.05
**SBP (mmHg)**	114.26	8.50
**DBP (mmHg)**	69.81	9.68
**AST (U/L)**	21.03	7.54
**ALT (U/L)**	23.82	16.28
**GGT (U/L)**	15.57	14.92
**Total cholesterol (mg/dl)**	171.92	28.08
**LDL-cholesterol (mg/dl)**	90.65	29.99
**HDL-cholesterol (mg/dl)**	53.15	16.95
**Triglycerides (mg/dl)**	88.24	38.97
**Triglycerides/HDL-ratio**	1.86	1.17
**Total cholesterol/HDL-ratio**	3.53	1.29
**Uric acid (mg/dl)**	5.04	1.19
**CRP (mg/dl)**	0.28	0.29
**HbA1c (%)**	5.28	0.33
**Fasting glucose (mg/dl)**	97.67	8.36
**2h-postprandial glucose (mg/dl)**	115.09	16.16
**Fasting insulin (μUI/ml)**	21.11	12.15
**2h-postprandial insulin (μUI/ml)**	119.98	90.82
**HOMA-IR**	5.17	3.15
**HOMA-B**	221.20	120.57
**IGI**	2.64	1.88
**Matsuda-index**	0.25	0.13
**AUC_g_**	249.40	29.49
**AUC_i_**	234.19	163.95
**AUC_i/_AUC_g_ ratio**	0.93	0.62
**FT4 (pmol/L)**	15.48	2.32
**TSH (uUI/ml)**	2.68	2.94
**Fasting Asprosin (pg/ml)**	374.28	77.23
**2h-postprandial Asprosin (pg/ml)**	375.27	81.26

BMI, Body mass index; SDS, standard deviation score; WC, waist circumference; WHtR, WC-to-height ratio; SBP, ; systolic blood pressure; DBP, diastolic blood pressure; ALT, alanine aminotransferase; AST, aspartate aminotransferase; GGT, Gamma-Glutamyl Transferase; HbA1c, glycated haemoglobin; 2h-postprandial glucose, 120-minutes OGTT glucose levels; 2h-postprandial insulin, 120-minutes OGTT insulin levels; 2h-postprandial asprosin, ; 120-minutes OGTT asprosin levels; HOMA-IR, model assessment of insulin resistance; HOMA-B, homeostasis model assessment for β-cell function; IGI, insulinogenic index; AUCg, area under the curve for glucose and AUCi, insulin; FT3, free triiodothyronine; FT4, free thyroxine; TSH, thyroid stimulating hormone.

As expected, a significant increase in blood glucose and insulin levels was documented from fasting to 2h-timepoint of OGTT (**Table 2**). Conversely, fasting and 2h-postprandial asprosin serum levels did not significantly differ in the entire study population (**Table 2**). The same trend of asprosin levels was confirmed considering sex and pubertal stage (**Table 2**). Categorizing patients according to the presence or not of IR, no significant change was documented between fasting and 2h-postprandial asprosin levels in either subgroup, as occurred by dividing the population in relation to BMI SDS or the presence of steatosis (**Table 2**). A trend for subjects with IFG to have higher 2h-postprandial asprosin levels was demonstrated, although this finding did not reach statistical significance (**Table 2**).

**Table 2:** Variations in blood glucose, insulin and asprosin between fasting and 2-h postprandial levels.

Subgroup (N° of patients)	Blood glucose			Insulin			Asprosin		
	T0	T120	p	T0	T120	p	T0	T120	p
**Entire study population(79)**	97.67 ± 8.36	115.08 ± 16.158	0.000	21.11 ± 12.15	119.97 ± 90.82	0.000	374.28 ± 77.23	375.27 ± 81.26	0.837
**Male (40)**	97.35 ± 7.52	116.85 ± 16.02	0.000	19.26 ± 11.59	118.97 ± 98.32	0.000	375.03 ± 80.86	370.27 ± 70.11	0.840
**Female (39)**	98 ± 9.23	113.28 ± 16.3	0.000	23.00 ± 12.56	121.01 ± 83.72	0.000	373.52 ± 74.37	380.41± 91.96	0.548
**Prepubertal (28)**	96 ± 8.19	119.21± 16.25	0.000	15.26 ± 8.20	119.51 ± 88.41	0.000	408.82 ± 111.14	395.04 ± 84.09	0.374
**Pubertal (51)**	98.59 ± 8.39	112.82 ± 15.81	0.000	24.32 ± 12.82	120.23 ± 92.99	0.000	355.33 ± 40.0	364.42 ± 78.39	0.265
**IR (53)**	99.56 ± 7.63	117.39 ± 15.29	0.000	26.26 ± 11.47	138.77 ± 102.37	0.000	382.37 ± 89.01	383.07 ± 94.54	0.968
**Non-IR (26)**	93.81 ± 8.59	110.38 ± 17.13	0.000	10.58 ± 3.89	81.65 ± 40.75	0.000	357.79 ± 41.34	359.37 ± 40.23	0.568
**Steatosis (31)**	97.9 ± 7.97	115.9 ± 15.98	0.000	24.65 ± 13.43	131.06 ± 102.88	0.000	368.55 ± 80.95	366.09 ± 68.18	0.984
**Non-steatosis (40)**	97.5 ± 8.68	116.03 ± 16.88	0.000	17.95 ± 10.68	108.29 ± 75.84	0.000	377 ± 79.92	379.79 ± 90.34	0.819
**BMI SDS** **>2.5 (65)**	97.05 ± 8.55	115.52 ± 16.83	0.000	21.16 ± 12.37	118.13 ± 89.86	0.000	378.93 ± 83.74	378.81 ± 86.12	0.959
**BMI SDS** **≤2.5 (14)**	100.57 ± 6.93	113.07 ± 12.89	0.000	20.85 ± 11.48	128.52 ± 98.16	0.000	352.70 ± 25.56	358.81 ± 52.4	0.551
**IFG (26)**	107.15 ± 5.70	119.34 ± 15.3	0.000	27.38 ± 12.13	136.05 ± 84.23	0.000	361.12 ± 39.23	382.91 ± 101.32	0.078
**Non-IFG (53)**	93.02 ± 4.71	113 ± 16.29	0.000	18.03 ± 11.01	112.09 ± 93.64	0.000	380.74 ± 89.8	371.52 ± 70.19	0.383

T0, Fasting time point of OGTT; T120, two-hour time point of OGTT. BMI, Body mass index; SDS, standard deviation score; IR, insulin resistance; IFG, impaired fasting glucose.

In particular, contrary to the expected physiological changes in asprosin levels in relation to the meal, the majority of patients in the entire study population (55.7%) had a significant increase in 2h-postprandial asprosin compared with fasting levels (349.38 ± 35.67 *vs* 380.75 ± 83.16; p=0.000), whereas in the remaining 44.3% of patients asprosin levels decreased (405.59 ± 101.30 vs 368.38 ± 79.47; p=0.000). Patients had a significant increase in 2h-postprandial asprosin had significantly higher fasting blood glucose levels and more frequently an IFG condition compared to patients who had a significant decrease in 2h-postprandial asprosin levels (**Table 3**).

**Table 3:** Comparison analysis between groups identified according to meal-related asprosin levels variation.

	Asprosin increase (N = 44 patients)	Asprosin decrease (N = 35 patients)	**p value**
**Age (years)**	11.90 ± 2.46	10.91 ± 2.72	0.11
**Sex (M/F)**	22/22	18/17	0.90
**Pubertal stage (prepubertal/pubertal)**	13/31	15/20	0.22
**BMI**	29.91 ± 4.04	28.98 ± 4.67	0.19
**BMI SDS**	3.08 ± 0.77	3.27 ± 1.09	0.47
**WHtR**	0.60 ± 0.06	0.59 ± 0.06	0.90
**SBP (mmHg)**	114.85 ± 8.12	113.48 ± 9.05	0.35
**DBP (mmHg)**	69.93 ± 9.10	69.65 ± 10.55	0.43
**AST (U/L)**	21.14 ± 8.70	20.89 ± 5.89	0.63
**ALT (U/L)**	25.25 ± 18.66	22.03 ± 12.73	0.66
**GGT (U/L)**	16.59 ± 19.89	14.37 ± 5.06	0.40
**Total cholesterol (mg/dl)**	171.20 ± 29.08	172.83 ± 27.16	0.89
**LDL-cholesterol (mg/dl)**	90.59 ± 30.99	90.73 ± 29.17	0.82
**HDL-cholesterol (mg/dl)**	52.63 ± 18.10	53.80 ± 15.65	0.75
**Triglycerides (mg/dl)**	88.29 ± 40.56	88.17 ± 37.46	0.77
**Triglycerides/HDL-ratio**	1.88 ± 1.26	1.82 ± 1.08	0.93
**Total cholesterol/HDL-ratio**	3.60 ± 1.52	3.43 ± 0.97	0.96
**Uric acid (mg/dl)**	5.16 ± 1.18	4.89 ± 1.21	0.22
**HbA1c (%)**	5.28 ± 0.32	5.29 ± 0.34	0.74
**Fasting glucose (mg/dl)**	99.25 ± 8.78	95.69 ± 7.46	0.04
**2h-postprandial glucose (mg/dl)**	112.98 ± 15.26	117.74 ± 17.07	0.23
**Fasting insulin (μUI/ml)**	21.59 ± 11.56	20.49 ± 12.99	0.41
**2h-postprandial insulin (μUI/ml)**	116.08 ± 81.19	124.88 ± 102.67	0.74
**HOMA-IR**	5.39 ± 3.18	4.88 ± 3.15	0.34
**HOMA-B**	215.32 ± 101.10	228.58 ± 142.54	0.85
**IGI**	2.79 ± 1.68	2.47 ± 2.11	0.24
**Matsuda-index**	0.24 ± 0.13	0.26 ± 0.14	0.55
**AUC_i/_AUC_g_ ratio**	0.91 ± 0.44	0.94 ± 0.79	0.50
**IR (yes/no)**	28/16	25/10	0.46
**IFG (yes/no)**	19/25	7/28	0.03
Steatosis (yes/no)*	17/23	14/17	0.82

Numerical data are expressed as mean ± standard deviation score. Categorical variables (sex, pubertal stage, IR, IFG, steatosis) are expressed as number of patients.

“Asprosin increase” group identified patients had a significant increase in 2h-postprandial asprosin compared with fasting levels. “Asprosin decrease” group identified patients had not a significant increase in 2h-postprandial asprosin compared with fasting levels.

*The presence of steatosis was evaluated in 71 patients.

BMI, Body mass index; WHtR, WC-to-height ratio; SBP, systolic blood pressure; DBP, diastolic blood pressure; ALT, alanine aminotransferase; AST, aspartate aminotransferase; GGT, Gamma-Glutamyl Transferase; HbA1c, glycated haemoglobin; 2h-postprandial glucose, 120-minutes OGTT glucose levels; 2h-postprandial insulin, 120-minutes OGTT insulin levels homeostasis; HOMA-IR, model assessment of insulin resistance; HOMA-B, homeostasis model assessment for β-cell function; IGI, insulinogenic index; AUCg, area under the curve for glucose; and AUCi, insulin; IR, insulin resistance; IFG, impaired fasting glucose.

Correlation analysis documented a significant positive relation between asprosin and lipid profile parameters. In particular, after adjustment for sex, pubertal stage and BMI SDS, fasting asprosin levels were correlated to triglycerides (r= 0.243, p= 0.036) and triglycerides/HDL-ratio (r= 0.244, p=0.035), while 2h-postprandial asprosin levels were correlated to LDL (r=0.292, p=0.011), triglycerides (r= 0.250, p= 0.031) and triglycerides/HDL-ratio (r= 0.232, p= 0.045). A correlation between asprosin and BMI, BMI SDS, and glucose metabolism parameters (HOMA-IR, HOMA-B, IGI, Matsuda index, AUC_i_/AUC_g_-ratio, OGTT insulin and blood glucose) was not documented (data not shown).

Considering that 55.7% of patients had an unexpected increase in 2h-postprandial asprosin compared to fasting levels, a stepwise multiple logistic regression analysis was applied to assess which metabolic variables significantly affected the asprosin increase condition. Asprosin level increase condition was significantly influenced by fasting glycaemia, HOMA-IR and HOMA-B. In particular, the asprosin level increase condition was significantly positively associated with HOMA-IR and fasting glycaemia and negatively associated with HOMA-B (**Table 4**). Conversely, no significant associations were found in the models that assessed the increase in asprosin with respect to parameters of lipid metabolism (Model 3), transaminases and the presence of hepatic steatosis (Model 4) (data not shown).

**Table 4:** Stepwise multiple logistic regression analysis of variables affecting serum asprosin levels.

Model 1: age, sex, BMI SDS, HOMA-IR, HOMA-B, IGI, Matsuda index, AUC_i_/AUC_g_-ratio										
	B		P		OR		95%		CI	
**HOMA-IR**	0.34		0.047		1.41		1.005		1.977	
**HOMA-B**	-0.01		0.035		0.99		0.984		0.999	
**Model 2: age, sex, BMI SDS, fasting and 2-hpostprandial glycaemia, fasting and 2-h postprandial insulin**										
	**B**		**P**		**OR**		**95%**		**CI**	
**Fasting glycaemia**	0.07		0.02		1.073		1.009		1.141

Dependent variable: asprosin increase/non-increase between 0 and 120 minutes OGTT time point evaluations.

BMI SDS, Body mass index standard deviation score; postprandial glucose, 120-minutes OGTT glucose levels; postprandial insulin, 120-minutes OGTT insulin level homeostasis; HOMA-IR, model assessment of insulin resistance; HOMA-B, homeostasis model assessment for β-cell function; IGI, insulinogenic index; AUCg, area under the curve for glucose; and AUCi, insulin; CI, Confidence interval.

Further logistic regression analysis documented a significant association between IFG and the increase in asprosin levels, even after adjustment for HOMA-IR, BMI SDS, sex, and pubertal stage (**Table 5**).

**Table 5:** OR and 95% Confidence interval (CI) for impaired fasting glucose according to asprosin increase.

Models	OR (95%CI)	*P value*
**1**	3.040 (1.095 8.436)	0.033
**2**	3.325 (1.049 10.545)	0.041
**3**	3.287 (1.033 10.458)	0.044
**4**	3.286 (1.033 10.457)	0.044
**5**	3.211 (1.006 10.249)	0.048

Dependent variable: asprosin increase between 0 and 120 minutes OGTT time point evaluations.

Model 1: crude model; Model 2: HOMA IR; Model 3: HOMA IR, BMI SDS; Model 4: HOMA IR, BMI SDS, sex; Model 5: HOMA IR, BMI SDS, sex, pubertal stage.

## Discussion

Asprosin is a glucogenic adipokine that stimulates hepatic glucose release *via* the cyclic-AMP (cAMP)-protein kinase A (PKA) pathway (4). Under physiological conditions, asprosin levels decrease after meal intake in both murine models and in humans (4).

For the first time, in our study we have demonstrated, differently than expected, no significant variation between fasting and 2-h postprandial asprosin levels in obese, non-diabetic children, suggesting an alteration in the meal-related regulation of asprosin secretion in these subjects. Furthermore, in the group of patients who had an increase in asprosin between fasting and 2-hour postprandial levels, the IFG condition was significantly more frequent than in patients who had a decrease in asprosin levels. HOMA-IR, fasting glycaemia and IFG significantly influenced the asprosin increase condition in 2-h postprandial evaluation, suggesting that IR and IFG may affect the physiological meal-related variation in asprosin levels.

IR plays a central role in the relationship between obesity and the associated risk of IGR, T2DM, metabolic syndrome and cardiovascular disease (1, 16, 21). Obese children with a more significant alteration in insulin sensitivity are at greater risk of developing T2DM and cardiovascular diseases, compared with peers without IR, given the same BMI (1, 16, 21). Asprosin may be involved in the pathogenesis of IR and IGR. Romere et al. first demonstrated elevated plasma asprosin levels in humans and mice with IR, increased levels of glucose and 2h-insulin in fast mice undergoing continuous or pulsatile overexpression of asprosin, and a significant decrease in glucose and insulin levels, secondary to reduced hepatic glucose release, after immunologic or genetic asprosin action inhibition (4). Several studies have shown higher serum asprosin levels in subjects with IR and/or T2DM and a correlation between these pathological conditions and asprosin serum levels (8–10, 12–14). Wang et al. documented higher plasma asprosin levels in IGR (including IFG and IGT subjects) and newly diagnosed T2DM patients compared to subjects with normal glucose regulation (NGR), showing a significant positive correlation between asprosin and HOMA-IR and a negative correlation between asprosin and HOMA-B (10). Moreover, these authors reported higher asprosin levels in a fasting state and at all intravenous glucose tolerance test (IVGTT) samplings, in adults with IGR compared to NGR and newly diagnosed T2DM, suggesting a role of asprosin as biomarkers to predict prediabetes (10). Interestingly, an alteration of meal-dependent circadian oscillation of asprosin serum levels has been reported in T2DM adult patients compared to non-diabetic controls (14); in particular, Zhang et al. documented a non-significant decrease of 2h-postprandial serum asprosin levels during an OGTT (14).

Our study found that asprosin levels did not significantly decrease at 2h-postprandial assessment in obese non-diabetic children and that this trend was significantly influenced by the presence of IR and IFG. We speculate that in obese children and adolescents with IR and IFG there may be an altered production of asprosin which in turn may promote IR worsening by stimulating hepatic glucose secretion and subsequent hyperinsulinemia. The asprosin increase condition and, in particular, alteration of its circadian secretion, might be an early biomarker of IGR in obese children with IR.

Asprosin may also be implicated in the pathogenesis of the well-known association between IR and lipid profile alteration (22). Zhang et al. demonstrated a strong correlation between asprosin and lipid metabolism (13). Similarly, in our study, fasting and 2h-postprandial asprosin levels significantly correlated with lipid profile parameters even after adjustment for sex, pubertal stage and BMI SDS.

Sample size represented a limitation of our study. For ethical reasons, it was not possible to have healthy children undergo an OGTT to perform the same assessments conducted on the study population. Data on liver ultrasound were available for 71 patients as 8 patients discontinued follow-up before undergoing ultrasound. Due to the cross-sectional design of the study, it is not possible to generalize the findings of the present study. On the other hand, our study has significant strengths. First, our study population consisted in a homogeneous sample of obese Caucasian children with equal distribution according to sex. Second, the assessment of both fasting and meal-related asprosin levels through standardizing the evaluations by performing an OGTT. This type of assessment, compared with a random asprosin measurement, allowed us to verify an alteration of the physiological meal-related fluctuation of asprosin levels in a pediatric population with obesity. The assay standardization is useful in the interpretation of results as it partly compensates for the lack of plasma reference values for asprosin.

In conclusion, this is the first study to demonstrate a non-significant variation between fasting and 2h-postprandial asprosin levels in obese, non-diabetic children and to document an influence of IR, fasting glycaemia and IFG on meal-related changes of asprosin serum levels. The alteration of asprosin circadian secretion might be an early biomarker of IGR in obese children with IR. Further studies on a larger study population are needed to confirm and verify these findings.

## Data Availability Statement

The raw data supporting the conclusions of this article will be made available by the authors, without undue reservation.

## Ethics Statement

The studies involving human participants were reviewed and approved by the ethics committee of Messina. Written informed consent to participate in this study was provided by the participants’ legal guardian/next of kin.

## Author Contributions

DC and MW contribute to conception and design of the study. DC, TA, GP, SC, and AL organized the database and prepared the tables. RI and MC performed asprosin measurements. AA performed statistical analysis. DC and MW wrote the first draft of the manuscript. MW and RI supervised the work. DC and MW wrote the final version of the manuscript. All authors contributed to manuscript revision, read, and approved the submitted version.

## Conflict of Interest

The authors declare that the research was conducted in the absence of any commercial or financial relationships that could be construed as a potential conflict of interest.

## Publisher’s Note

All claims expressed in this article are solely those of the authors and do not necessarily represent those of their affiliated organizations, or those of the publisher, the editors and the reviewers. Any product that may be evaluated in this article, or claim that may be made by its manufacturer, is not guaranteed or endorsed by the publisher.
